# Lymphoid Tissue Mesenchymal Stromal Cells in Development and Tissue Remodeling

**DOI:** 10.1155/2016/8419104

**Published:** 2016-04-13

**Authors:** Luca Genovese, Andrea Brendolan

**Affiliations:** Division of Experimental Oncology, IRCCS San Raffaele Scientific Institute, 20132 Milan, Italy

## Abstract

Secondary lymphoid organs (SLOs) are sites that facilitate cell-cell interactions required for generating adaptive immune responses. Nonhematopoietic mesenchymal stromal cells have been shown to play a critical role in SLO function, organization, and tissue homeostasis. The stromal microenvironment undergoes profound remodeling to support immune responses. However, chronic inflammatory conditions can promote uncontrolled stromal cell activation and aberrant tissue remodeling including fibrosis, thus leading to tissue damage. Despite recent advancements, the origin and role of mesenchymal stromal cells involved in SLO development and remodeling remain unclear.

## 1. Introduction

Secondary lymphoid organs (SLOs) such as spleen and lymph nodes (LNs) play a critical role in host defense. This function is ensured by the unique cellular composition of lymphoid tissues characterized by the presence of stationary mesenchymal stromal cells and highly motile hematopoietic cells. Although most of the attention has been concentrated on hematopoietic cells and their functions, the stromal counterpart has recently emerged as an important player in regulating immune responses and tissue homeostasis [1]. Alterations in stromal cell composition and function have been associated with different pathological conditions such as autoimmunity, infections, and cancer. Despite recent advances in the field, little is known about the origin and nature of the different mesenchymal stromal cells involved in tissue remodeling during homeostasis and disease. Indeed, a better understanding of the cells and signals contributing to tissue remodeling will provide basic knowledge for designing strategies aiming to promote tissue repair during pathological conditions such as chronic inflammation. Here we discuss the different steps involved in the maturation of lymphoid tissue mesenchymal stromal cells and how these cells contribute to tissue remodeling during normal and pathological conditions.

## 2. Development of Secondary Lymphoid Tissues and Origin of Stromal Diversity

Development of SLOs is spatiotemporally regulated during embryogenesis and requires interaction between lymphoid tissue stromal organizer (LTo) cells of mesenchymal origin and lymphoid tissue inducer (LTi) cells derived from the hematopoietic lineage [2–4]. The interaction between these two-cell types occurs through engagement of several molecules including the lymphotoxin *β* receptor (LT*β*R) expressed on mesenchymal cells by lymphotoxin *α*1*β*2 (LT*αβ*) expressed on hematopoietic cells. LTi cells, which belong to the family of type 3 innate lymphoid cells, are also characterized by expression of CD45, CD4, interleukin-7 receptor *α* (IL-7R*α*), integrin *α*4*β*7, receptor-activator of NF-*κ*B (RANK/TRANCE-R), and the chemokine receptor CXCR5. Conversely, mesenchymal stromal cells express, in addition to LT*β*R, platelet-derived grow factor-receptor *α* (PDGFR*α*) and the chemokine CXCL13 [5]. The latter is a critical signal for attracting LTi cells expressing the CXCL13-receptor CXCR5 to the site of organ formation [3]. Although differences exist in the initial steps of spleen and lymph node development, lymphomesenchymal interactions are critical to promote the differentiation of mesenchymal progenitors to mature stromal cells and the establishment of distinct tissue compartments. Studies on mice deficient for molecules expressed by LTi (e.g., CXCR5) or by LTo cells (e.g., CXCL13, LT*β*R) have shown defects ranging from organ agenesis to disrupted tissue architecture [3]. Although the developmental relationship between embryonic mesenchymal cells of the lymphoid tissue anlage and the adult stromal compartment is not fully elucidated, recent findings demonstrated that spleen stromal cells arise from multipotent embryonic mesenchymal cells of the Nkx2-5^+^Isl1^+^ lineage [6]. It was shown that nearly all mature mesenchymal stromal cells, namely, follicular dendritic cells (FDCs) of the B-cell follicle, marginal reticular cells (MRCs) localized underneath the marginal sinus, fibroblastic reticular cells (FRCs) in the T-cell zone, and NG2+ perivascular cells, originate from embryonic mesenchymal descendants [6]. While this mechanism for generating stromal diversity applies to the spleen, the embryonic lineages contributing to the different stromal cells of the LN remain unclear. Furthermore, Nkx2-5^+^Isl1^+^ mesodermal precursors do not contribute to spleen or lymph node endothelial cells, thus indicating that different mesodermal lineages are involved in generating SLO stromal diversity including lymphatic and endothelial cells. Interestingly, endothelial cells have been shown to undergo endothelial-mesenchymal transition (EndMT) during cardiac development [7, 8]. Whether this mechanism also contributes to the generation of stromal diversity during SLO development remains an open question.

Stromal cells express several receptors of TNF superfamily of proteins including LT*β*R, RANK, and Tumor Necrosis Factor Receptors [9]. By engaging with their ligands, LT*αβ* and Tumor Necrosis Factor (TNF) expressed by hematopoietic cells, these receptors trigger the secretion of homeostatic chemokines such as CCL19/CCL21 and CXCL13 that play a critical role in attracting and positioning T- and B-cells within SLOs [9]. Indeed, mice deficient for LT*β*R or genes encoding chemokines secreted by stromal cells have profound disorganization of the white pulp area and defective immune functions, demonstrating the critical role played by mesenchymal stromal cells as “organizers” of the lymphoid compartments [10]. Stromal cells also produce the extracellular matrix (ECM), a tridimensional framework of reticular fibers composed of basement membrane and interstitial matrix components that provide structural support [11]. In the T-cell zone, FRCs form the so-called conduit system, a reticular collagenous network that allows the transport and distribution of small molecules or particles from the periphery to T-cell zone [12].

In the B-cell follicle, FDCs play a crucial role in promoting B-cell immunity [13]. FDCs promote recruitment of B-lymphocytes into the follicles through secretion of CXCL13 that binds CXCR5 expressed on B-cells. This stromal cell-type presents antigens in the form of immune complexes that are bound via Fc and complement receptors, thus stimulating B-cells through the B-cell receptor (BCR) and promoting germinal center formation. Generation of FDC networks relies on TNFR and LT*β*R signaling; however, only signals through LT*β*R were shown to be required for FDC maintenance [14]. MRCs are stromal cells that localize underneath the marginal sinus and in the outermost region of the follicle and express CXCL13 and MAdCAM-1 [15]. Although the exact function of MRCs remains elusive, recent work showed that MRCs contribute to the accumulation of FDC during germinal center formation [16]. In addition, the expression of B-cell chemokines and the close association of this cell-type with CD169^+^ marginal metallophilic macrophages suggest their possible involvement in supporting local niches.

## 3. The Extracellular Matrix of Secondary Lymphoid Organs

The stroma is defined as the connective and functionally supportive structure of a tissue or organ. It consists of fibroblasts and vascular cells and their associated extracellular matrix (ECM) proteins such as collagens, fibronectin, glycosaminoglycans, and proteoglycans [17]. The ECM has been viewed only as a tridimensional framework to which cells adhere. However, work over the past years has demonstrated that the ECM is not merely an inactive player in tissue homeostasis, but, instead, a structure with define physical and biochemical properties able to affect cell behavior [11]. Indeed, the continuous cell-ECM cross talk allows cells to sense the surrounding environment, resulting in changes in gene expression. For instance, the ECM affects cell behavior by different mechanisms: (i) by regulating cell-accessibility to growth factors; (ii) by providing cells with ligands for cell-surface receptors; (iii) and by affecting migration and proliferation through ECM-stiffness and composition [18, 19]. Deregulation in ECM structure and composition has been associated with different pathological conditions including tissue fibrosis and cancer by promoting apoptotic evasion, cell survival, proliferation, and invasion [18, 20, 21].

In peripheral lymphoid tissues, two biochemically and morphologically different ECMs exist: the interstitial matrix (IM) and the basement membrane (BM). The IM represents the ECM that connect fibroblastic reticular cells and is composed of interstitial collagens (types I, III, V, and XI) that confer high flexibility and tensile strength, as well as proteoglycans and glycoproteins, such as fibronectin, tenascin, and vitronectin, able to recognize and bind several cytokines, chemokine, and growth factors [17, 19]. The BM is a sheet of ECM that acts primarily to separate the different functional compartments of the organ. It is mainly composed of four molecules: type IV collagen, noncollagenous glycoproteins belonging to the family of laminins, heparan sulphate proteoglycans, and glycoproteins [17, 19, 22]. One of the peculiar three-dimensional structures of SLOs is the conduit system, a complex structure of FRCs and reticular fibers that promotes the rapid transport of small molecules, such as chemokines, cytokines, and small molecular weight antigens, from peripheral sites to the lymphoid compartments [23]. The conduit also acts as a scaffold for lymphocyte locomotion within SLOs, thus facilitating cell distribution and interactions [24]. The reticular fibers of the conduits show a highly organized core of collagens, mostly type I and type III, and associated with fibrils ensheathed by the BM. The latter is composed of laminin isoforms 511, 411, and 332, heparan sulphate proteoglycan, perlecan, collagen type IV, and nidogen to which FRCs adhere [25–28]. Collagen IV can bind several chemokines and cytokines such as CCL21 and IL7 produced by FRC, thus facilitating the positioning of T lymphocytes within SLOs. FRCs are interconnected and ensheathing the conduit system in which dendritic cells (DCs) fill the free space and pick up antigens directly from the conduit [29, 30]. This means that lymphocytes are not in direct contact with the basal membrane and the fluid present in the conduit, though antigens and small molecules are accessible through FRCs or DCs present in the gaps of the conduit. Recently, it has been demonstrated that the specific expression of perifollicular laminin *α*5 in the marginal zone (MZ) of the spleen drives the localization of a specialized B-cell population expressing integrin *α*6*β*1 to this area. Moreover, laminin *α*5 was found to regulate not only the localization but also the fate and long-term survival including the antibody responses of MZ B-cells. These findings indicate that stromal-derived ECM actively influences immune cell behavior through several mechanisms [18, 19].

## 4. Remodeling of the Stromal Microenvironment in Acute Inflammation 

The acute phase of an adaptive immune response is characterized by lymph node expansion in order to host the incoming wave of naïve lymphocytes and the proliferation of antigen-specific lymphocytes prior to returning to its physiological size during the resolution phase [31–33]. In this process, the distribution of stromal cells and their associated ECM undergoes transient changes to support immune responses. These include the expansion of fibroblastic reticular and lymphatic networks and the increase in size and permeability of high endothelial venues (HEVs) and lymphatic vessels in order to facilitate the extensive accumulation of naïve lymphocytes and fluid from the periphery [34–37]. Although the origin and nature of stromal cells that participate in LN hypertrophy and remodeling remain elusive, recent studies have identified FRCs as key players in the process [38]. Stretch of preexisting FRC networks and FRC proliferation are involved in LN enlargement [33, 36, 39]. Dendritic cells (DCs) have been shown to regulate the stretch of FRCs, via CLEC-2 on DC binding to podoplanin (PDPN) on FRC and resulting in the inhibition of PDPN-mediated FRC contractility, and relaxation of the stromal networks [31, 32]. Changes in FRC contractility could directly influence FRC proliferation through mechanotransduction, a process known to convert mechanical forces into chemical or genetic changes at cellular level [32, 40]. The nature of inflammatory stimuli affects the timing at which the proliferation of FRC occurs. Indeed, whereas LPS stimulates FRC proliferation as early as 24 hrs after injection, immunization with ovalbumin (OVA) in complete Freund's adjuvant (CFA) or Montanide causes stromal cells to proliferate modestly within 2 days and more vigorously until day 5 after injection [31, 33, 41]. The initial phase of proliferation is dependent on CD11c^+^ DC, whereas T- and B-cells contribute to the subsequent expansion phase [33, 41]. The findings that ablation of LT*β*R signaling in stromal cells abrogated FRC proliferation indicate that LT*αβ* from lymphocytes plays, at least in part, a role in remodeling of the FRC network [41, 42]. Interestingly, inflammation following CFA immunization causes changes in stromal composition and gene expression within T-cell zone stromal cells. It was reported that inflamed B-cell follicles extend towards the T-cell zone and induce the expression of CXCL13, a chemokine normally produced by FDCs, in stromal cells. Induction of CXCL13 was shown to depend on LT*αβ* from B-cells and the cells induced to express CXCL13 were called versatile stromal cells (VSC) [43]. Interestingly, during the contraction phase of B-cell follicles, VSCs downregulate CXCL13 expression, thus indicating a degree of plasticity of this mesenchymal cell type. From a developmental perspective, the origin and nature of VSCs remain unknown as the signaling underlying their plasticity [43].

Many viral infections induce a generalized immunosuppression that could be transient, during the acute phase, or prolonged, in chronic viral infections. In the case of lymphocytic choriomeningitis virus (LCMV), it was shown that infected FRCs are killed by LCMV-specific CD8^+^ T-cells during the acute phase of infection. Loss of the FRCs appears to be mediated by perforin-dependent and perforin-independent mechanisms and strongly correlates with the impairment of CCL19 and CCL21 expression, two chemokines important for positioning T-cells within the FRC zone [1, 44]. Interestingly, remodeling and restoration of stromal network integrity occurs approximately four weeks after LCMV infection and depends, at least in part, on LTi-stroma interactions via LT*β*R signaling [44]. The survival and proliferation of adult LTi cells are induced by IL-7. Stromal and lymphatic endothelial cells expressing IL-7 are critical during LN remodeling after LCMV infection, as demonstrated by the findings that ablation of IL-7 expressing stromal cells strongly impairs restoration of tissue integrity [45]. In the spleen, regeneration of the stromal network was shown to depend on local Nkx2-5^+^Islet1^+^ mesenchymal descendants, possibly possessing stem cell activity. In this setting, local expansion of mesenchymal stromal cells and not migration of peripheral cells appeared to be the underlying mechanism of tissue regeneration [6]. Nevertheless, the exact nature of mesenchymal stromal cells involved in tissue repair after LCMV infection remains unclear. Perivascular cells have been proposed to act as mesenchymal stem cells during tissue repair and thus could represent good candidates in SLO remodeling after loss of tissue integrity.

## 5. Persistent Stromal Remodeling and Tissue Fibrosis during Chronic Inflammation

Chronic inflammation is characterized by persistent inflammatory stimuli or by deregulation of the mechanisms involved in the resolution phase [9]. This condition stimulates uncontrolled stromal cell activation and consequent aberrant tissue remodeling including fibrosis [9, 46]. One example is the chronic infection caused by human immunodeficiency virus-1 (HIV-1). Indeed, a large number of patients infected with HIV-1 have profound lymphoid tissue disorganization and show limited or absent immune reconstitution despite suppression of replicating virus in plasma [47–49].

Immunohistological studies have demonstrated that acute HIV-1 infection is associated with generalized lymph node enlargement. Moreover, abnormal LN architecture is associated with progressive loss of immune responses and correlates with disease progression, culminating in end-stage AIDS [50–54]. Furthermore, several observations describe an inverse correlation between the number of CD4^+^ T-cells in the LN paracortical region and tissue fibrosis in HIV-1 infected patients [55, 56]. In the case of nonhuman primates (NHP) infected with simian immunodeficiency virus (SIV), the accumulation of T_reg_ cells expressing transforming growth factor *β*1 (TGF*β*1) correlates with the pathological deposition of fibrotic collagen by T-cell zone mesenchymal stromal cells. Indeed, T_reg_ cells were shown to secrete TGF*β*1 and stimulate resident fibroblasts to produce procollagen and chitinase 3-like-1 (CHI3L1), an enzyme involved in the maturation of procollagen and fibrosis [42, 57, 58]. This increased and uncontrolled deposition of fibrotic ECM strongly affects the capacity of the T-cells to recognize the prosurvival factor IL-7 produced by FRCs. This mechanism seems to explain the high degree of apoptosis and the depletion of both naïve CD4^+^ and CD8^+^ T-cells (the latter are not usually infected by HIV-1) occurring in infected patients [42, 59]. On the other hand, the survival of FRCs depends on LT*αβ* from T-cells [42], and the increase in T-cell apoptosis causes a reduction of LT*αβ* that ultimately results in loss of FRC networks and, consequently, the prosurvival signal IL-7 [42, 60, 61]. The reciprocal interactions between the FRCs and T-cells have been recently demonstrated in mice upon LT*β*R-Ig treatment, to deplete the FRC networks, or anti-CD3 administration, to induce T-cell apoptosis. Indeed, mice with a depleted FRC network have reduced T-cells, and vice versa mice depleted of T-cells have lost FRC networks [42, 62]. In addition to LT*β*R, TNF is also involved in the maintenance of FRC, as demonstrated by reduced lymphoid tissue fibrosis in NHP treated with anti-TNF antibody [63].

Given the important role of the FRC network in lymphocytes locomotion, loss of it has an effect on T-cell migration within the LNs. Thus, a vicious cycle of progressive destruction of the LN architecture ultimately limits the possibility of restoring normal immune responses, despite suppression of replicating virus in the plasma [47–49]. It remains unclear whether stromal cell subsets other than FRC contribute to fibrosis and if this process could be reverted by pharmacological means. Endothelial cells have been implicated in tissue fibrosis, though it is unknown if this lineage is involved in remodeling the lymphoid stromal microenvironment through endomesenchymal transition during chronic inflammation [64].

In addition to FRCs, progressive loss of the FDC networks has been also described in HIV-1 infection. As a consequence, B-cell specific immune responses to HIV-1 and other pathogens are compromised [65]. The finding that FDC networks are present in HIV-1 infected patients after 2.5 years of antiretroviral therapy, with a pattern similar to the one shown in SLO from healthy volunteers, indicates that tissue remodeling and repair of follicular stromal cell are reversible. However, it is not clear whether changes in FDCs correlate with fibrosis or are directly linked to the viral load [66]. Nevertheless, the cellular mechanism involved in restoration of FDC networks upon treatment remains unclear.

## 6. Conclusion

**Figure 1:**
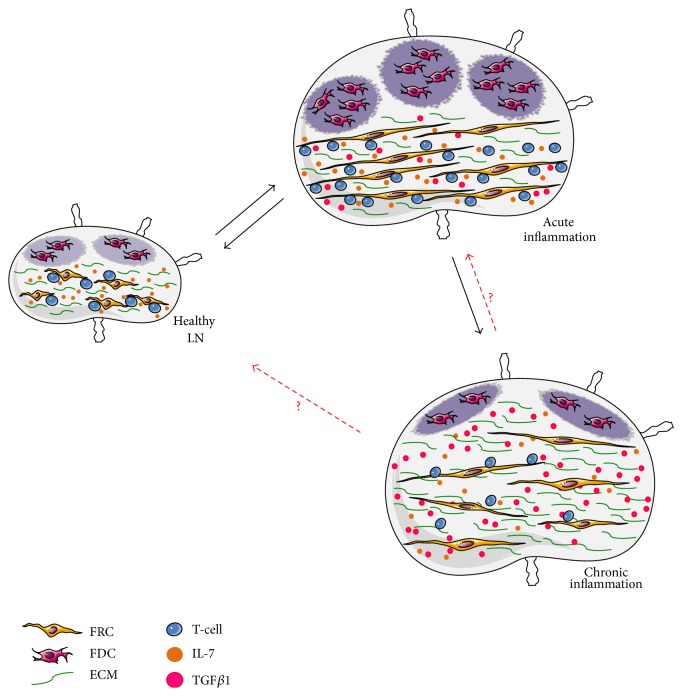


Secondary lymphoid organs represent the primary site for initiating and developing adaptive immune responses, as well as for maintenance of lymphocyte homeostasis. During inflammation, the stromal microenvironment undergoes profound remodeling to support immune responses and mesenchymal stromal cells are emerging as important players (Figure 1). A better understanding of the nature of mesenchymal stromal cells involved in lymphoid tissue remodeling together with knowledge on the signaling networks contributing to stromal cell activation and proliferation will help to identify novel targets and design new strategies in order to prevent tissue damage and to restore integrity upon injury.

## References

[1] Mueller S. N., Germain R. N. (2009). Stromal cell contributions to the homeostasis and functionality of the immune system. *Nature Reviews Immunology*.

[2] Brendolan A., Rosado M. M., Carsetti R., Selleri L., Dear T. N. (2007). Development and function of the mammalian spleen. *BioEssays*.

[3] Brendolan A., Caamaño J. H. (2012). Mesenchymal cell differentiation during lymph node organogenesis. *Frontiers in Immunology*.

[4] Mebius R. E. (2003). Organogenesis of lymphoid tissues. *Nature Reviews Immunology*.

[5] Bénézech C., White A., Mader E. (2010). Ontogeny of stromal organizer cells during lymph node development. *Journal of Immunology*.

[6] Castagnaro L., Lenti E., Maruzzelli S. (2013). Nkx2–5^+^islet1^+^ mesenchymal precursors generate distinct spleen stromal cell subsets and participate in restoring stromal network integrity. *Immunity*.

[7] Pucéat M. (2013). Embryological origin of the endocardium and derived valve progenitor cells: from developmental biology to stem cell-based valve repair. *Biochimica et Biophysica Acta*.

[8] Medici D., Kalluri R. (2012). Endothelial-mesenchymal transition and its contribution to the emergence of stem cell phenotype. *Seminars in Cancer Biology*.

[9] Buckley C. D., Barone F., Nayar S., Bénézech C., Caamaño J. (2015). Stromal cells in chronic inflammation and tertiary lymphoid organ formation. *Annual Review of Immunology*.

[10] Mackay F., Majeau G. R., Lawton P., Hochman P. S., Browning J. L. (1997). Lymphotoxin but not tumor necrosis factor functions to maintain splenic architecture and humoral responsiveness in adult mice. *European Journal of Immunology*.

[11] Lokmic Z., Lämmermann T., Sixt M., Cardell S., Hallmann R., Sorokin L. (2008). The extracellular matrix of the spleen as a potential organizer of immune cell compartments. *Seminars in Immunology*.

[12] Mebius R. E., Kraal G. (2005). Structure and function of the spleen. *Nature Reviews Immunology*.

[13] Allen C. D. C., Cyster J. G. (2008). Follicular dendritic cell networks of primary follicles and germinal centers: phenotype and function. *Seminars in Immunology*.

[14] Aguzzi A., Kranich J., Krautler N. J. (2014). Follicular dendritic cells: origin, phenotype, and function in health and disease. *Trends in Immunology*.

[15] Katakai T. (2012). Marginal reticular cells: a stromal subset directly descended from the lymphoid tissue organizer. *Frontiers in Immunology*.

[16] Jarjour M., Jorquera A., Mondor I. (2014). Fate mapping reveals origin and dynamics of lymph node follicular dendritic cells. *The Journal of Experimental Medicine*.

[17] Sorokin L. (2010). The impact of the extracellular matrix on inflammation. *Nature Reviews Immunology*.

[18] Lu P., Weaver V. M., Werb Z. (2012). The extracellular matrix: a dynamic niche in cancer progression. *Journal of Cell Biology*.

[19] Song J., Lokmic Z., Lämmermann T. (2013). Extracellular matrix of secondary lymphoid organs impacts on B-cell fate and survival. *Proceedings of the National Academy of Sciences of the United States of America*.

[20] Genovese L., Zawada L., Tosoni A. (2014). Cellular localization, invasion, and turnover are differently influenced by healthy and tumor-derived extracellular matrix. *Tissue Engineering Part A*.

[21] Lu P., Takai K., Weaver V. M., Werb Z. (2011). Extracellular matrix degradation and remodeling in development and disease. *Cold Spring Harbor Perspectives in Biology*.

[22] Timpl R. (1996). Macromolecular organization of basement membranes. *Current Opinion in Cell Biology*.

[23] Nolte M. A., Beliën J. A. M., Schadee-Eestermans I. (2003). A conduit system distributes chemokines and small blood-borne molecules through the splenic white pulp. *Journal of Experimental Medicine*.

[24] Bajénoff M., Egen J. G., Koo L. Y. (2006). Stromal cell networks regulate lymphocyte entry, migration, and territoriality in lymph nodes. *Immunity*.

[25] Sixt M., Kanazawa N., Selg M. (2005). The conduit system transports soluble antigens from the afferent lymph to resident dendritic cells in the T cell area of the lymph node. *Immunity*.

[26] Gretz J. E., Anderson A. O., Shaw S. (1997). Cords, channels, corridors and conduits: critical architectural elements facilitating cell interactions in the lymph node cortex. *Immunological Reviews*.

[27] Kaldjian E. P., Elizabeth Gretz J., Anderson A. O., Shi Y., Shaw S. (2001). Spatial and molecular organization of lymph node T cell cortex: a labyrinthine cavity bounded by an epithelium-like monolayer of fibroblastic reticular cells anchored to basement membrane-like extracellular matrix. *International Immunology*.

[28] Karttunen T., Sormunen R., Risteli L., Risteli J., Autio-Harmainen H. (1989). Immunoelectron microscopic localization of laminin, type IV collagen, and type III pN-collagen in reticular fibers of human lymph nodes. *Journal of Histochemistry and Cytochemistry*.

[29] Gretz J. E., Kaldjian E. P., Anderson A. O., Shaw S. (1996). Sophisticated strategies for information encounter in the lymph node: the reticular network as a conduit of soluble information and a highway for cell traffic. *Journal of Immunology*.

[30] Roozendaal R., Mebius R. E., Kraal G. (2008). The conduit system of the lymph node. *International Immunology*.

[31] Astarita J. L., Cremasco V., Fu J. (2015). The CLEC-2-podoplanin axis controls the contractility of fibroblastic reticular cells and lymph node microarchitecture. *Nature Immunology*.

[32] Acton S. E., Farrugia A. J., Astarita J. L. (2014). Dendritic cells control fibroblastic reticular network tension and lymph node expansion. *Nature*.

[33] Chyou S., Benahmed F., Chen J. (2011). Coordinated regulation of lymph node vascular-stromal growth first by CD11c + cells and then by T and B cells. *Journal of Immunology*.

[34] Webster B., Ekland E. H., Agle L. M., Chyou S., Ruggieri R., Lu T. T. (2006). Regulation of lymph node vascular growth by dendritic cells. *Journal of Experimental Medicine*.

[35] Girard J.-P., Moussion C., Förster R. (2012). HEVs, lymphatics and homeostatic immune cell trafficking in lymph nodes. *Nature Reviews Immunology*.

[36] Tan K. W., Yeo K. P., Wong F. H. S. (2012). Expansion of cortical and medullary sinuses restrains lymph node hypertrophy during prolonged inflammation. *Journal of Immunology*.

[37] Angeli V., Ginhoux F., Llodrà J. (2006). B cell-driven lymphangiogenesis in inflamed lymph nodes enhances dendritic cell mobilization. *Immunity*.

[38] Fletcher A. L., Acton S. E., Knoblich K. (2015). Lymph node fibroblastic reticular cells in health and disease. *Nature Reviews Immunology*.

[39] Katakai T., Hara T., Sugai M., Gonda H., Shimizu A. (2004). Lymph node fibroblastic reticular cells construct the stromal reticulum via contact with lymphocytes. *Journal of Experimental Medicine*.

[40] Wozniak M. A., Chen C. S. (2009). Mechanotransduction in development: a growing role for contractility. *Nature Reviews Molecular Cell Biology*.

[41] Yang C.-Y., Vogt T. K., Favre S. (2014). Trapping of naive lymphocytes triggers rapid growth and remodeling of the fibroblast network in reactive murine lymph nodes. *Proceedings of the National Academy of Sciences of the United States of America*.

[42] Zeng M., Smith A. J., Wietgrefe S. W. (2011). Cumulative mechanisms of lymphoid tissue fibrosis and T cell depletion in HIV-1 and SIV infections. *Journal of Clinical Investigation*.

[43] Mionnet C., Mondor I., Jorquera A. (2013). Identification of a new stromal cell type involved in the regulation of inflamed B cell follicles. *PLoS Biology*.

[44] Scandella E., Bolinger B., Lattmann E. (2008). Restoration of lymphoid organ integrity through the interaction of lymphoid tissue-inducer cells with stroma of the T cell zone. *Nature Immunology*.

[45] Onder L., Narang P., Scandella E. (2012). IL-7-producing stromal cells are critical for lymph node remodeling. *Blood*.

[46] Nebuloni M., Zawada L., Ferri A. (2013). HIV-1 infected lymphoid organs upregulate expression and release of the cleaved form of uPAR that modulates chemotaxis and virus expression. *PLoS ONE*.

[47] Haase A. T. (1999). Population biology of HIV-1 infection: viral and CD4+ T cell demographics and dynamics in lymphatic tissues. *Annual Review of Immunology*.

[48] Martín M., Echevarría S., Leyva-Cobián F., Pereda I., López-Hoyos M. (2001). Limited immune reconstitution at intermediate stages of HIV-1 infection during one year of highly active antiretroviral therapy in antiretroviral-naive versus non-naive adults. *European Journal of Clinical Microbiology and Infectious Diseases*.

[49] Gea-Banacloche J. C., Clifford Lane H. (1999). Immune reconstitution in HIV infection. *AIDS*.

[50] Biberfeld P., Ost A., Porwit A. (1987). Histopathology and immunohistology of HTLV-III/LAV related lymphadenopathy and AIDS. *Acta Pathologica Microbiologica et Immunologica Scandinavica A*.

[51] Ioachim H. L., Cronin W., Roy M., Maya M. (1990). Persistent lymphadenopathies in people at high risk for HIV infection. Clinicopathologic correlations and long-term follow-up in 79 cases. *American Journal of Clinical Pathology*.

[52] Pantaleo G., Graziosi C., Demarest J. F. (1994). Role of lymphoid organs in the pathogenesis of human immunodeficiency virus (HIV) infection. *Immunological Reviews*.

[53] Schacker T., Collier A. C., Hughes J., Shea T., Corey L. (1996). Clinical and epidemiologic features of primary HIV infection. *Annals of Internal Medicine*.

[54] Tindall B., Barker S., Donovan B. (1988). Characterization of the acute clinical illness associated with human immunodeficiency virus infection. *Archives of Internal Medicine*.

[55] Schacker T. W., Nguyen P. L., Beilman G. J. (2002). Collagen deposition in HIV-1 infected lymphatic tissues and T cell homeostasis. *Journal of Clinical Investigation*.

[56] Diaz A., Alós L., León A. (2010). Factors associated with collagen deposition in lymphoid tissue in long-term treated HIV-infected patients. *AIDS*.

[57] Estes J. D., Wietgrefe S., Schacker T. (2007). Simian immunodeficiency virus-induced lymphatic tissue fibrosis is mediated by transforming growth factor *β*1-positive regulatory T cells and begins in early infection. *Journal of Infectious Diseases*.

[58] Estes J. D., Li Q., Reynolds M. R. (2006). Premature induction of an immunosuppressive regulatory T cell response during acute simian immunodeficiency virus infection. *Journal of Infectious Diseases*.

[59] Paiva D. D., Morais J. C., Pilotto J., Veloso V., Duarte F., Lenzi H. L. (1996). Spectrum of morphologic changes of lymph nodes in HIV infection. *Memorias do Instituto Oswaldo Cruz*.

[60] Estes J. D., Haase A. T., Schacker T. W. (2008). The role of collagen deposition in depleting CD4+ T cells and limiting reconstitution in HIV-1 and SIV infections through damage to the secondary lymphoid organ niche. *Seminars in Immunology*.

[61] Zeng M., Southern P. J., Reilly C. S. (2012). Lymphoid tissue damage in HIV-1 infection depletes naïve T cells and limits T cell reconstitution after antiretroviral therapy. *PLoS Pathogens*.

[62] Zeng M., Haase A. T., Schacker T. W. (2012). Lymphoid tissue structure and HIV-1 infection: life or death for T cells. *Trends in Immunology*.

[63] Tabb B., Morcock D. R., Trubey C. M. (2013). Reduced inflammation and lymphoid tissue immunopathology in rhesus macaques receiving anti-tumor necrosis factor treatment during primary simian immunodeficiency virus infection. *Journal of Infectious Diseases*.

[64] Zeisberg E. M., Tarnavski O., Zeisberg M. (2007). Endothelial-to-mesenchymal transition contributes to cardiac fibrosis. *Nature Medicine*.

[65] Fauci A. S. (1993). Multifactorial nature of human immunodeficiency virus disease: implications for therapy. *Science*.

[66] Zhang Z.-Q., Schuler T., Cavert W. (1999). Reversibility of the pathological changes in the follicular dendritic cell network with treatment of HIV-1 infection. *Proceedings of the National Academy of Sciences of the United States of America*.

